# Postischemic Long-Term Treatment with Qiangli Tianma Duzhong Capsule Improves Brain Functional Recovery via the Improvement of Hemorrheology and the Inhibition of Platelet Aggregation in a Rat Model of Focal Cerebral Ischemia

**DOI:** 10.1155/2013/795365

**Published:** 2013-10-29

**Authors:** Li-Zhi Hong, Wei-wei Gu, Yong Ni, Min Xu, Lei Yang, Yan-Li Liu, Shi-Ling Yang, Qiang Zhou, Xiu-Mei Gao, Hui-Ling Zhang

**Affiliations:** ^1^Department of Pharmacology and Laboratory of Cerebrovascular Pharmacology, College of Pharmaceutical Science, Soochow University, Suzhou 215123, China; ^2^ChunKe Guiyang Pharmaceutical R & D Co., Ltd., Guiyang 550018, China; ^3^Xiyuan Hospital, China Academy of Chinese Medical Sciences, Beijing 100091, China

## Abstract

Qiangli Tianma Duzhong capsule (TMDZ), a Chinese herbal drug, is clinically used to improve functional outcome in patients with ischemic stroke in China. This study was conducted to establish whether postischemic long-term treatment with TMDZ could reduce the loss of injured hemisphere and confer the improvements of neurological outcome in chronic survival of rats with 2 h middle cerebral artery occlusion (MCAO)/reperfusion brain injury and its primary mechanisms. We found that TMDZ (44.5, 89, or 178 mg/kg), administered per os 6 h after the onset of ischemia and for 28 consecutive days, significantly improved the behavior deficits, beginning on day 7, and further improved later. TMDZ treatment also markedly reduced the tissue loss of the injured hemisphere and improved histopathology. In the meantime, TMDZ treatment could improve hemorrheology and inhibit platelet aggregation. These results provide the first evidence that post-ischemic long-term treatment with TMDZ confers the improvements of neurological outcome and the loss of injured hemisphere in an animal ischemic stroke model, and its mechanisms might be associated with the improvements of hemorrheology and the inhibition of platelet aggregation.

## 1. Introduction

Cerebral ischemia or stroke, one of the leading causes of death and long-term disability in aged populations, often results in irreversible brain damage and subsequent loss of neuronal function. The medications commonly used for stroke can be divided into four groups: thrombolytic agents, antiplatelet agents, anticoagulants, and neuroprotective agents [1]. The only approved stroke medication, tissue plasminogen activator, is a thrombolytic that targets the thrombus in the blood vessel. Neuroprotective agents, which may make the brain more resistant to damage from stroke, have generated much interest as another approach to stroke treatment. To date, however, neuroprotective agents have not reached routine clinical use and remain less than ideal [2].

In recent years, much attention has been paid to traditional herbal medicines [3–7]. Qiangli Tianma Duzhong capsule (TMDZ) mainly comprises traditional herbal medicines, Tianma and Duzhong. From the view of traditional medicine, the function of TMDZ is promoting blood circulation, removing blood stasis, and relaxing the muscles pain and has been used in the treatment of ischemic stroke in China. So far, clinical studies have demonstrated a beneficial effect of TMDZ in functional recovery in patients with stroke. However, the details of functional recovery including characterization of how anatomical and histological recovery changes following brain ischemia are influenced by treatment with TMDZ, and its mechanisms are largely absent in the literature. Here, we demonstrated that after ischemia administrated for 4 weeks, TMDZ can reduce the loss of damaged hemisphere and improve brain histopathology and neurological outcome, and its mechanisms might be associated with the improvements of hemorrheology and the inhibition of platelet aggregation in a rat model of 2 h MCAO and reperfusion.

## 2. Materials and Methods

### 2.1. Animals and Treatments

Sprague Dawley (SD) male rats (280–310 g) were purchased from the Center for Experimental Animals, Soochow University (certificate no. 20020008, Grade II). They were housed four per cage in a standard animal room with a 12 h light/dark cycle and given free access to food and water. NIH guidelines for the care and use of laboratory animals were followed in all animal procedures.

Qiangli Tianma Duzhong capsule (TMDZ) was provided by ChunKe Guiyang Pharmaceutical R & D Co., Ltd. TMDZ 44.5, 89, or 178 mg/kg (dissolved indistilled water) or vehicle (distilled water) was administrated per os 6 h after the onset of ischemia and consecutive 28 days after ischemia. Sham-operated or ischemic-reperfusion (IR) control animals received vehicle (distilled water) per os.

### 2.2. Rat Models of 2 h Middle Cerebral Artery Occlusion (MCAO) and Reperfusion

Rats (*n* = 10/group) were anesthetized with intraperitoneal injection of 4% choral hydrate (350 mg/kg). Through a ventral midline incision, the right common carotid artery (CCA), external carotid artery (ECA), and internal carotid artery (ICA) were isolated, and ECA and CCA were ligated. A 30 mm length of monofilament nylon suture (Φ0.22–0.24 mm), with its tip rounded by heating near a flame, was inserted from the right CCA to ICA through a small incision in the common carotid artery and then advanced to the Circle of Willis to occlude the origin of the right middle cerebral artery for 2 hours, and then the suture was withdrawn. Body temperature was closely monitored with a rectal probe and maintained in the range of 37.0 ± 0.5°C with a heating pad (Institute of Biomedical Engineering, CAMS, BME-412A ANIMAL REGULATOR, 308005669) during and after surgery until recovery from anesthesia. Sham-operated rats underwent the same procedures except for cutting a small incision and inserting a monofilament nylon suture to the artery. Behavioral tests were evaluated before stroke and at 2 h ischemia and 4 h, 1, 3, 7, 14, 21, and 28 days of reperfusion. Body weight was measured every week. For the observation of the brain damage, animals were killed, and the brains were dissected and sliced in a plastic module (Harvard Apparatus, 3 mm thickness) 28 days after ischemia. For morphology analysis, animals were sacrificed 28 days after ischemia (*n* = 3/group) by transcardial perfusion of 0.9% normal saline, followed by 4% paraformaldehyde in 100 mM phosphate buffer. The brains were then fixed, embedded with paraffin. Brain coronal sections at the level of the caudate putamen which showed typical infarction were selected and sectioned to 10 *μ*m, and then brain coronal sections were stained with hematoxylin-eosin (HE). The pyramidal cortical cells were examined with a microscopy. For quantification of cells, 10 microscopic fields (magnification 20x) in each section across ischemic cortical regions in the ipsilateral hemisphere were analyzed. Three sections were used for each animal. The number of cells in each field was counted by an examiner who was blinded to the experimental conditions [8].

### 2.3. Behavioral Testing

Neurological deficits were examined at 2 h ischemia and 4 h reperfusion using a 5-point scale adapted and modified from Zhang et al. [9]. Specifically, no neurological deficit equals 0; right Horner's syndrome counts 1 point; failure to extend left forelimb and hindlimb counts 1 point each; turning to left counts 1 point; and circling to left counts 1 point.

### 2.4. Asymmetry in the Use of Forelimbs for Postural Support (Cylinder Test)

Cylinder Tests were performed 1, 3, 7, 14, 21, and 28 days after ischemia reperfusion. Briefly, animals were placed into a plexiglass cylinder, and their behavior was observed for forelimb use asymmetry during vertical movements along the wall of the cylinder. The final score was calculated as (nonimpaired forelimb movement − impaired forelimb movement)/(nonimpaired forelimb movement + impaired forelimb movement + both movements), as previously described in the rat [10]. A total of 20 movements were recorded during the 10 min test.

### 2.5. Asymmetry-Corner Test

Corner tests were performed 1, 3, 7, 14, 21, and 28 days after ischemia reperfusion. Briefly, in the home cage, an animal was placed between the two angled boards. When entering deep into the corner, both sides of the vibrissae are stimulated together. The animal then rears forward and upward then turns back to face the open end. Twenty trials were performed for each rat, and the percentage of right turns versus left turns was calculated. Only turns involving full rearing along either boards were recorded [11, 12].

### 2.6. Magnetic Resonance Image (MRI) Analysis for Lesion Size

Rats were anesthetized with isoflurane, were placed in an animal holder/MRI probe apparatus, and were positioned inside the magnet. The animal's head was held in place inside the imaging coil. All MRI measurements were performed using a 7 Teslar, 18 cm bore superconducting magnet (Oxford Magnet Technologies) interfaced to a UNITYINOVA console (Oxford Instruments, UK, and Varian Inc., Palo Alto, CA, USA). T_2_-weighted images (T_2_WI) were obtained from a 1.0 mm thick coronal section with a 0.5 mm gap using a 30 mm × 30 mm field of view, TR = 3000 ms, TE = 37 ms, and b value = 0 and reconstructed using a 256 × 256 image matrix. Accurate positioning of the brain was performed to center the image slice 5 mm posterior to the rhinal fissure with the head of the rat held in a flat skull position. For each slice, the higher intensity lesions in T_2_WI were marked as the ischemic lesion area [13]. MRI measurements were obtained 28 days after MCAO (*n* = 3 for each group).

### 2.7. Determination of Hemorrheology

Blood samples were taken from the abdominal aorta of rats and were mixed with Heparin Li for blood viscosity and plasma viscosity, 3.2% citric acid for FBG, EDTA·K_2_ for HCT, and 3.2% sodium citrate for ESR. Blood viscosity was measured with a cone-plate viscometer (Brookfield Engineering Laboratories, Inc., Stoughton, MA, USA). Plasma viscosity (PV) was centrifuged at 3000 rpm for 15 min in a centrifuge to obtain the plasma. The plasma viscosity was measured by an Automatic Blood Rheometer LBY-N6B (Beijing Precil Instrument CO., LTD, Beijing, China). Fibrinogen (Fb), heamatocrit (HCT), and erythrocyte sedimentation rate (ESR) were detected with a laser-assisted automatic hemorheological analyzer (modified MDK-B100, MDK, Inc., Chongqing, China). All measurements were conducted at 4°C.

### 2.8. Platelet Aggregation

Rat platelet suspensions were prepared as previously described [14]. In brief, blood was collected from rats with or without stroke which had taken TMDZ for 4 weeks and was mixed with 3.2% citrate for ADP or EDTA K_2_ for thrombin. Platelet-rich plasma (PRP) was prepared by centrifugation at 500 rpm for 3 min at room temperature, and platelet aggregation induced by ADP (4 *μ*mol/mL, Solarbio) was measured using an Aggregometer (TYXN-96, Shanghai General Machine Electricity Technological Research Institute, Shanghai, China). For thrombin, The PRP was then centrifuged at 1500 rpm for 5 min, and the resulting platelet pellet was resuspended in the washing buffer (NaCl 140 mM, KCl 2.7 mM, NaH_2_PO_4_·2H_2_O 0.4 mM, NaHCO_3_ 12 mM, MgCl_2_·6H_2_O 1 mM, glucose 5 mM, HEPES 10 mM, PGE1 100 nM, and BSA 3.5 mg/mL, pH 6.6) and then centrifuged at 1000 rpm for 5 min. The platelet was resuspended in the suspension buffer (NaCl 140 mM, KCl 2.7 mM, NaH_2_PO_4_·2H_2_O 0.4 mM, NaHCO_3_ 12 mM, MgCl_2_·6H_2_O 1 mM, glucose 5 mM, HEPES 10 mM, and BSA 3.5 mg/mL, pH 7.4), and the platelet aggregation induced by thrombin (3 U/mL, Sigma) was measured.

### 2.9. Statistical Analysis

Statistical analysis was carried out by one-way analysis of variance (ANOVA) followed by a post hoc Tukey test. Differences were considered significant when *P* < 0.05.

## 3. Results

### 3.1. Qiangli Tianma Duzhong Capsule (TMDZ) Increases Body Weight

**Figure 1:**
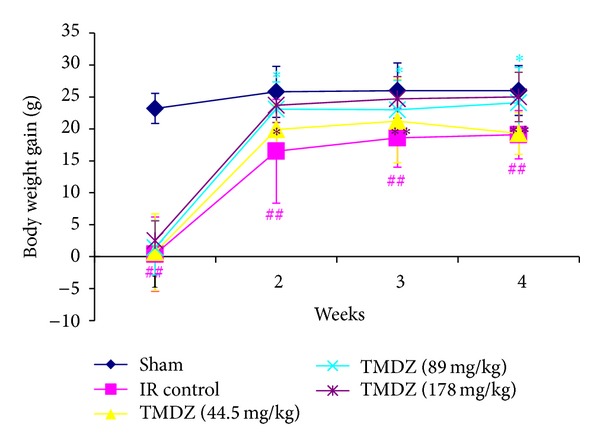
Post-ischemic long-term treatment with TMDZ increases the body weight after ischemic stroke. Animals underwent 2 h MCAO and reperfusion and received either an oral administration of TMDZ or vehicle (distilled water) at 2 h MCAO, 4 h reperfusion, and every day later for 28 days, and the body weight was measured every week. Compared to rats in ischemia-reperfusion control (IR Control), TMDZ treatment significantly increased body weight from day 7 (the second week), which persisted throughout the additional 3-week survival period. Values shown are mean ± S.D., *n* = 10. Statistical analysis was carried out with one-way ANOVA followed by a post hoc Tukey test. ^##^ *P* < 0.01 versus sham-operated group (Sham); *P < 0.05 and **P < 0.01 versus ischemia-reperfusion control group.

Compared to the rats in ischemia-reperfusion control (IR control), TMDZ treatment significantly increased body weight from day 7 (the second week), which persisted throughout the 4-week survival period (Figure 1).

### 3.2. TMDZ Reduces Brain Loss and Lesion Size

**Figure 2:**
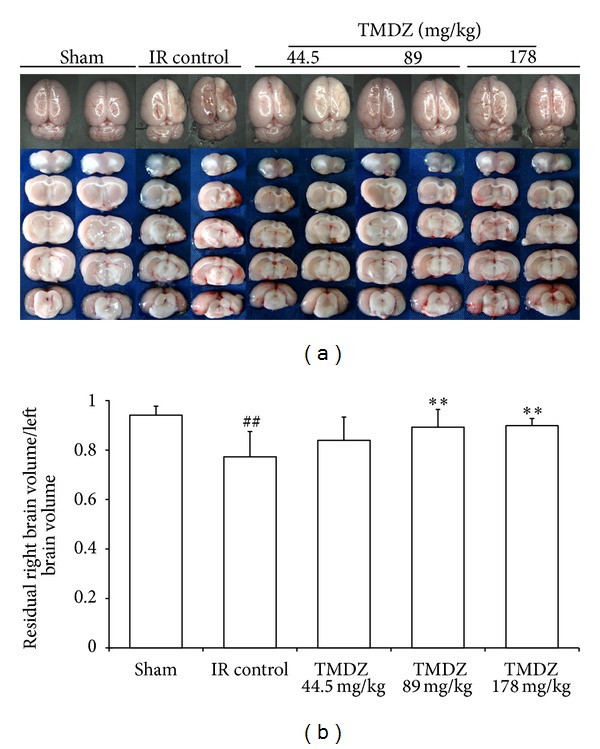
Post-ischemic long-term treatment with TMDZ reduces brain tissue loss after ischemic stroke. Animals underwent 2 h MCAO and reperfusion and received either an oral administration of TMDZ or vehicle (distilled water) at 2 h MCAO, 4 h reperfusion, and every day later for 28 days. The representative images of the whole brain and the sliced brain (a). The volume of right hemisphere (injured hemisphere) was indicated as the percentage of ipsilateral hemisphere volume of the contralateral hemisphere volume (indirect volume calculation) (b). Values shown are mean ± S.D., *n* = 10. Statistical analysis was carried out with one-way ANOVA followed by a post hoc Tukey test. ^#^ *P* < 0.05 and ^##^ *P* < 0.01 versus sham-operated group (Sham); **P < 0.01 versus ischemic-reperfusion control group (IR Control).

**Figure 3:**
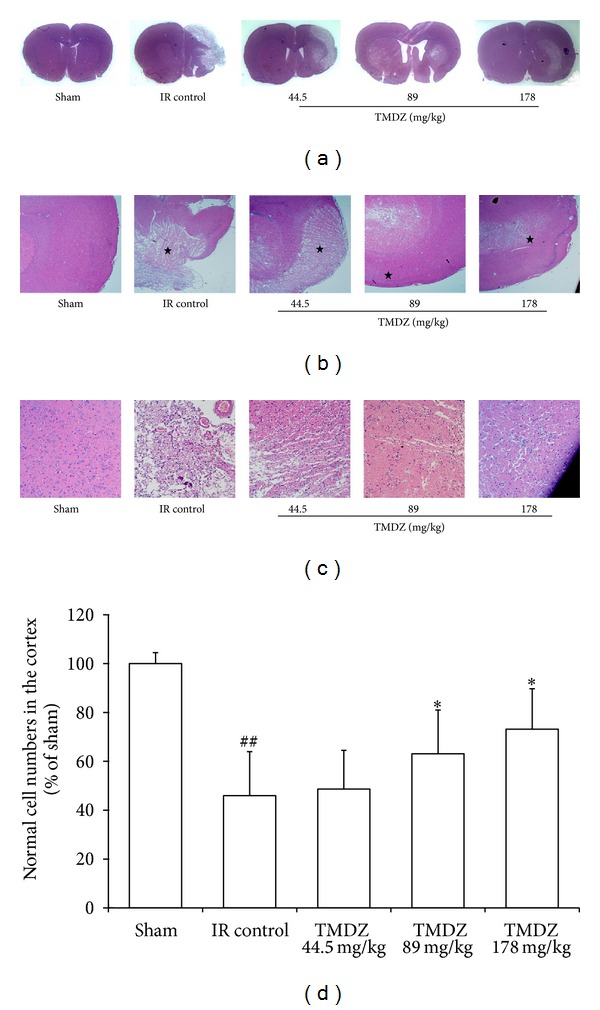
Post-ischemic long-term treatment with TMDZ improves the histopathological changes after ischemic stroke. Animals underwent 2 h MCAO and reperfusion and received either an oral administration of TMDZ or vehicle (distilled water) at 2 h MCAO, 4 h reperfusion, and every day later for 28 days, and the HE staining was measured 28 days after stroke. Computer generated MosaiX processed images (Carl Zeiss MicroImaging, Inc, Thornwood, NY, USA) of HE paraffin-embedded brain sections at coronal level (bregma +1.2 mm) from rats treated with vehicle or treated with TMDZ. The representative images of the entire slice (a), 4x (b), and 20x (c). The vehicle-treated rat (ischemia-reperfusion control group, IR Control) showed typical appearance of cystic necrosis, and pannecrosis involved the entire neocortical thickness, extending to subjacent regions. In contrast, rats treated with TMDZ showed less extensive damage. Values shown are mean ± S.D., *n* = 6. Statistical analysis was carried out with one-way ANOVA followed by a post hoc Tukey test. ^##^ *P* < 0.01 versus sham-operated group (Sham); **P < 0.01 versus ischemia-reperfusion control group (IR Control).

To reduce errors associated with processing tissue for histological analysis, the residual brain volume is presented as the percentage of ipsilateral (right) hemisphere volume of the contralateral (left) hemisphere volume (indirect volume calculation). The residual brain volumes (the integrated right hemisphere volume) after a 4-week stroke were reduced compared with the sham-operated hemisphere or the contralateral hemisphere in both ischemia-reperfusion control and TMDZ-treated groups. However, compared with the residual brain volume in ischemia-reperfusion control rats, TMDZ treatment significantly increased the residual brain volumes after consecutive administration for 4 weeks (Figure 2). Being consistent with these results, HE staining analysis also found that ischemia-reperfusion control rats showed ipsilateral-hemisphere tissue loss and extensive zones of cystic necrosis. In contrast, rats treated with TMDZ showed less extensive cortical and subcortical damage (Figure 3). Treatment with TMDZ significantly increased the cortical cells compared to the vehicle-treated rats (Figure 3).

**Figure 4:**
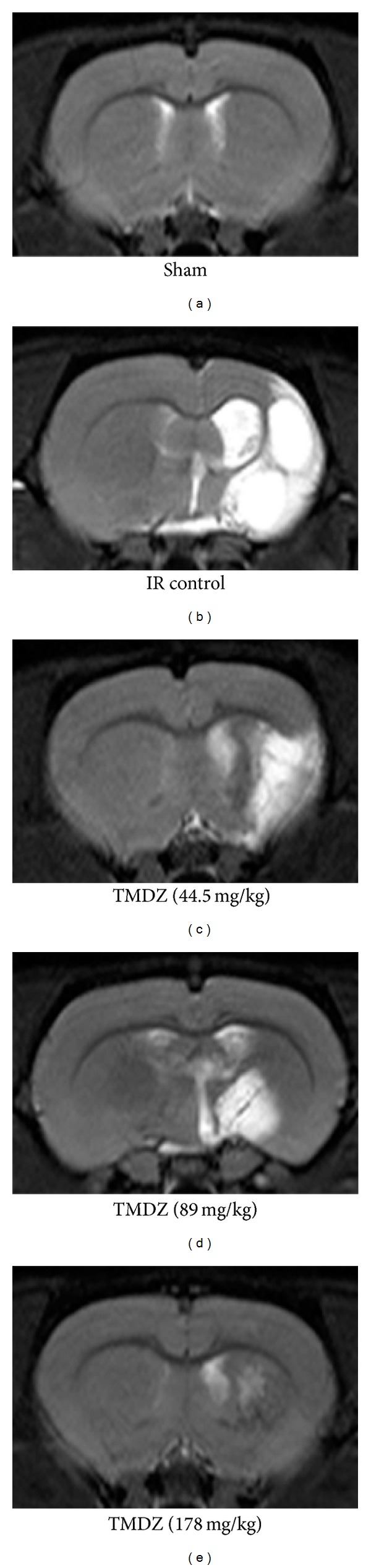
Post-ischemic long-term treatment with TMDZ reduces ischemic lesion with MRI. Animals underwent 2 h MCAO and reperfusion and received either an oral administration of TMDZ or vehicle (distilled water) at 2 h MCAO, 4 h reperfusion, and every day later for 28 days. T_2_-weighted images were obtained from experimental animals 28 days after MCAO and TMDZ were administrated.

In addition, the ischemic lesion size was estimated using an in vivo MRI. T_2_WIs were obtained 28 days after TMDZ or vehicle consecutive treatment. Coronal forebrain sections were obtained at the level of caudate-putamen complex (Figure 4). Lesion size (high intensity areas) was less in TMDZ-treated rats as compared to ischemia-reperfusion control rats (Figure 4).

These results suggest that post-ischemic long-term treatment with TMDZ reduces brain damage and brain tissue loss after stroke.

### 3.3. TMDZ Improves Neurological Outcome

**Figure 5:**
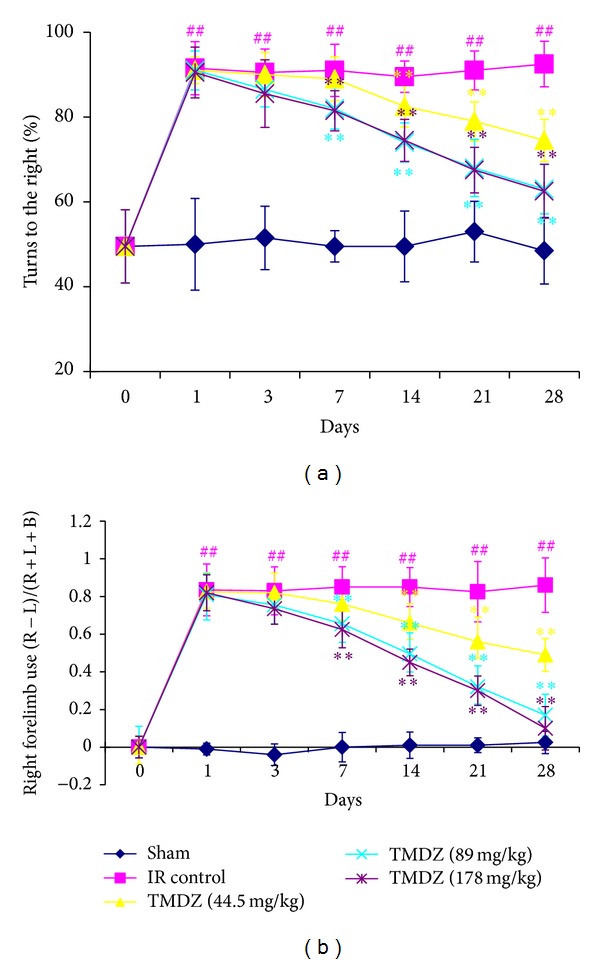
Post-ischemic long-term treatment with TMDZ improves neurological outcome after ischemic stroke. Animals underwent 2 h MCAO and reperfusion and received either an oral administration of TMDZ or vehicle (distilled water) at 2 h MCAO, 4 h reperfusion, and every day later for 28 days followed by neurological testing at 1, 3, 7, 14, 21, and 28 days. (a) The corner test demonstrated preferential turning to the right in animals that had undergone right MCAO/reperfusion and vehicle administration after stroke. Deficits persisted up to 28 days after stroke. Animals that received TMDZ, however, showed reduction of deficits over time. (b) The cylinder test demonstrated preferential right forearm placement in animals that had undergone right MCAO/reperfusion and vehicle administration after stroke. Deficits persisted up to 28 days after stroke. Animals that received TMDZ, however, showed reduction of deficits over time. Values shown are mean ± S.D., *n* = 10. Statistical analysis was carried out with one-way ANOVA followed by a post hoc Tukey test. ^##^ *P* < 0.01 versus sham-operated group (Sham); **P < 0.01 versus ischemic-reperfusion control group (IR Control).

Neurological score was normal in all animals before MCAO (score, 0). High-grade contralateral deficits (score, 2–5, Table 1) were presented at 2 h MCAO and 4 h reperfusion in all rats, and there was no significant difference among the groups (Table 1). After TMDZ consecutive treatment for 7 days, rats in TMDZ group showed a greater functional recovery than rats in ischemia-reperfusion control group and conferred a further improvement with time during the additional 3-week survival period in the corner test (Figure 5(a)) and cylinder test (Figure 5(b)). There were no adverse behavioral side effects observed with TMDZ administration. These results suggest that post-ischemic long-term treatment with TMDZ improves neurological outcome after stroke.

### 3.4. TMDZ Improves Hemorrheology

TMDZ did not change any of the hemorrheology parameters after normal rats were administrated with TMDZ for 4 weeks (Table 2). In rats, 4 weeks after stroke, the whole blood viscosity (WBV) of low shear rate, moderate shear rate, and high shear rate, the whole blood reduced viscosity (WBRV) of low shear rate, moderate shear rate, and high shear rate, and fibrinogen (Fb) were much higher as compared to sham-operated rats (Table 3). In contrast, TMDZ treatment for 4 weeks significantly decreased the WBV, WBRV, and Fb (Table 3). In addition, stroke induced an increase in plasma viscosity (PV), heamatocrit (HCT), erythrocyte sedimentation rate (ESR), erythrocyte sedimentation rate equation K value (ESRK), red blood cell aggregation index (RBCAI), and red blood cell rigidity index (RBCRI), but these parameters did not reach a statistically significant level. However, TMDZ obviously reduced the stroke induced increases in PV, HCT, RBCAI, and RBCRI (Table 3). There were no significant differences in the red blood cell deformation index (RBCDI) and the red blood cell electrophoresis index (RBCEI) among all groups (Table 3).

### 3.5. TMDZ Inhibits Platelet Aggregation

**Figure 6:**
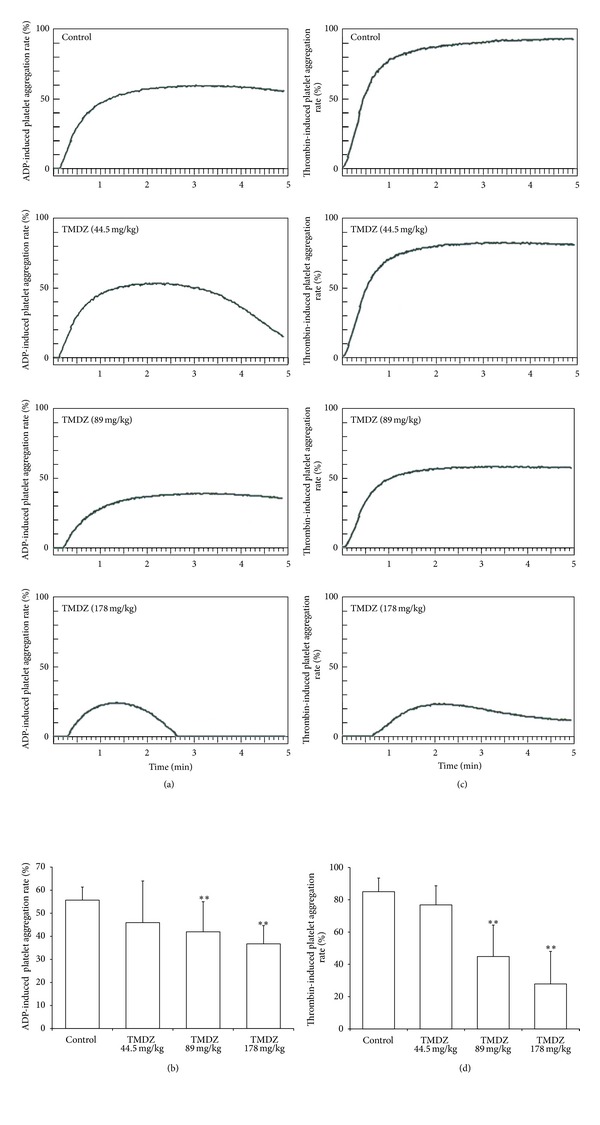
Long-term treatment with TMDZ inhibits ADP- or thrombin-induced platelet aggregation in normal rats. Animals received an oral administration of TMDZ or vehicle (distilled water) for 28 days, and then the blood was obtained, and the platelet aggregation induced by ADP or thrombin was detected. (a) and (c): representative curve of platelet aggregation induced by ADP (a) or thrombin (c). (b) and (d): quantitative analysis of changes in the platelet aggregation from 10 independent experiments of (a) and (c), respectively. Values shown are mean ± S.D., *n* = 10. Statistical analysis was carried out with one-way ANOVA followed by a post hoc Tukey test. ^##^ *P* < 0.01 versus vehicle-treated group (Control group).

**Figure 7:**
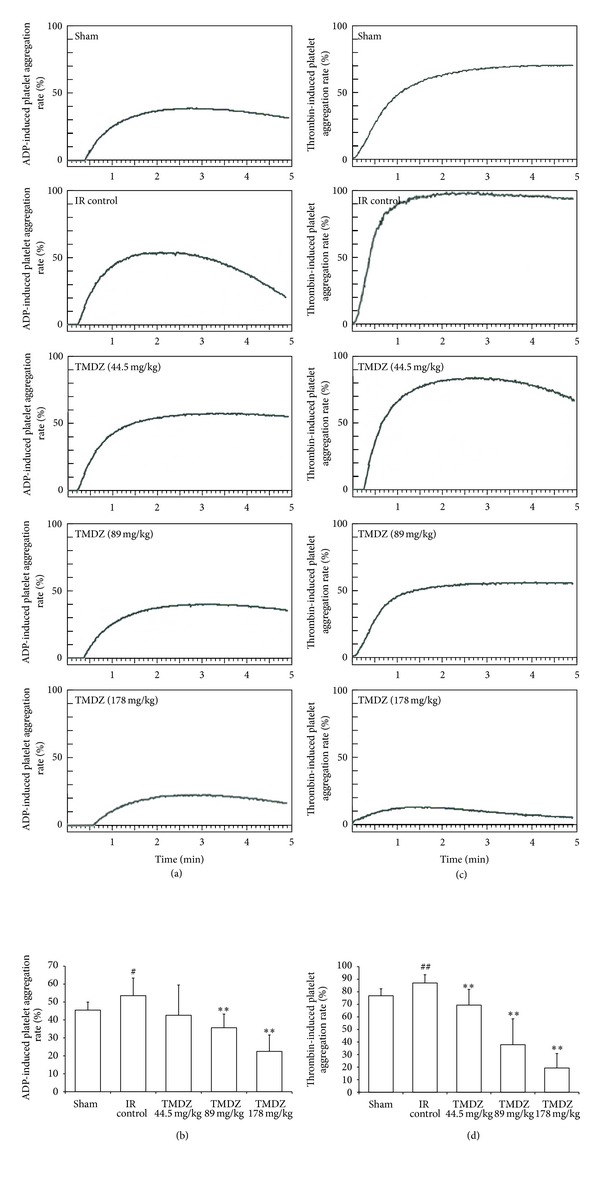
Long-term treatment with TMDZ inhibits ADP- or thrombin-induced platelet aggregation in ischemic stroke rats. Animals underwent 2 h MCAO and reperfusion and received either an oral administration of TMDZ or vehicle (distilled water) at 2 h MCAO, 4 h reperfusion and every day later for 28 days, and then the blood was obtained and the platelet aggregation induced by ADP or thrombin was detected. (a) and (c): representative curve of platelet aggregation induced by ADP (a) or thrombin (c). (b) and (d): quantitative analysis of changes in the platelet aggregation from 10 independent experiments of (a) and (c), respectively. Values shown are mean ± S.D., *n* = 10. Statistical analysis was carried out with one-way ANOVA followed by a post hoc Tukey test. ^#^ *P* < 0.05 and ^##^ *P* < 0.01 versus sham-operated group (Sham); **P < 0.01 versus ischemia-reperfusion control group (IR Control).

TMDZ significantly decreased the in vitro ADP- or thrombin-induced platelet aggregation after normal rats were administrated with TMDZ for 4 weeks (Figure 6). Furthermore, the in vitro ADP- or thrombin-induced platelet aggregation in ischemia-reperfusion control rats was dramatically increased, and TMDZ treatment for 4 weeks significantly decreased the ischemia-reperfusion-induced increase in platelet aggregation (Figure 7). These results suggest that TMDZ has a strong inhibitory effect on the platelet aggregation.

## 4. Discussion

Ischemic hypoxic brain injury often causes irreversible brain damage and permanent behavioral performance deficits. A well-controlled animal model of MCAO in rats, which produces consistent cortical and subcortical infarcts, closely resembles the large hemispheric infarcts resulting from proximal MCAO in patients [15]. Most of the studies published so far have focused on the fact that histopathology changes in the damaged brain occur in acute period of ischemic stroke, and more recently, much attention has also been paid to the histopathology changes in the damaged that brain occur in subacute and remote period of ischemic stroke. It has been demonstrated that hemispheric edema will happen to in the acute ischemic stroke, whereas brain tissue loss and atrophy will occur in the ischemic hemisphere in the subacute and remote ischemic stroke and the volume of injured hemisphere is largely reduced as compared to the contralateral hemisphere [16, 17], which was also confirmed in our current study 4 weeks after stroke in a rat model of 2 h MCAO and reperfusion. As assessed by both neurobehavioral and histological methods, this study demonstrates that consecutive oral administration of Qiangli Tianma Duzhong capsule (TMDZ) for 4 weeks after cerebral infarction results in a reduction in brain tissue loss and ischemic lesion size estimated from brain slices measurements and MRI analysis, an increase in body weight, and an improvement in behavioral performance and histopathology from HE analysis.

At present, the remarkable effect of TMDZ is not largely understood. An elevated blood viscosity value has been demonstrated in patient after both acute cerebral ischemia (24 h after the onset of stroke) and remote cerebral ischemic episode (3–6 months after the onset of stroke) although there are some differences in hemorheological parameters between patient with acute cerebral ischemia and patient with remote cerebral ischemic episode [18, 19]. Therefore, clinically, one of the drug therapies in patient with ischemic stroke is modifying the hemorrheological properties and leading to a decrease in blood viscosity, and thus resulting in better perfusion of brain and promotion of the recovery of functional deficits after stroke. Consistent with the pieces of literature [18, 19], in the present study, we also confirmed a significant increase in the whole blood viscosity (WBV) of low shear rate, moderate shear rate, and high shear rate and the whole blood reduced viscosity (WBRV) of low shear rate, moderate shear rate, and high shear rate 4 weeks after stroke in a rat model of 2 h MCAO and reperfusion. Blood viscosity depends on many factors. The most important are hematocrit value, plasma viscosity, and elasticity of the red cells and their aggregability [18]. The increased blood viscosity in the current study may result from a stroke that induced some tendency towards higher value in hematocrit value, plasma viscosity and the aggregability of the red blood cells (statistically not significant), and lower value in the elasticity of the red blood cells (statistically not significant). TMDZ treatment for 4 weeks after stroke significantly decreased the WBV and WBRV and also simultaneously decreased plasma viscosity (PV), heamatocrit (HCT), red blood cell aggregation index (RBCAI), and red blood cell rigidity index (RBCRI), which may contribute to the fact that TMDZ induced a decrease in blood viscosity. In addition, we found that stroke induced a high fibrinogen level, which is in agreement with the observation in a patient by Velcheva and Nikolova [19], and TMDZ decreased it.

The cascade of events leading to neuronal injury and death in ischemia includes the release of cytokines, free radicals, and platelet activation [20, 21]. The participation of activated platelets has been observed in brain microvessels of the ischemic microvascular bed after experimental middle cerebral artery occlusion (MCAO) [21]. Microvascular thrombi continue to accumulate even after recanalization of the MCAO, contributing to postischemic hypoperfusion and ongoing neuronal damage [22]. Thus, platelet aggregation may play a crucial role in MCAO-induced cerebral damage. In the present study, we showed that the platelet aggregation was significantly increased in ischemic rats 4 weeks after stroke by using an in vitro ADP- or thrombin-induced platelet aggregation and TMDZ treatment for 4 weeks significantly decreased the ischemia-induced increase in platelet aggregation. In addition, TMDZ also markedly decreased the platelet aggregation after normal rats were administrated with TMDZ for 4 weeks.

In summary, the present results firstly established that post-ischemic long-term treatment with TMDZ confers the improvements of neurological outcome and the loss of injured hemisphere in animal ischemic stroke models, and its mechanisms might be associated with the improvements of hemorrheology and the inhibition of platelet aggregation. These results are very encouraging for the wide application of TMDZ in patients with ischemic stroke. Whether post-ischemic long-term treatment with TMDZ produces neuroregeneration in the treatment of ischemic stroke remains to be further investigated in the near future.

## Figures and Tables

**Table 1:** Neurological scores after stroke. Neurological function was measured by 5-point test after 2 h MCAO and 4 h reperfusion. High-grade contralateral deficits were presented at 2 h MCAO and 4 h reperfusion in all rats, and there was no significant difference among the groups.

Group	Ischemia/reperfusion	Tianma Duzhong capsule		
	Control	44.5 mg/kg	89 mg/kg	178 mg/kg
Score	2.5 ± 0.71	2.4 ± 0.52	2.4 ± 0.52	2.4 ± 0.70

**Table 2:** Hemorrheology parameters in normal rats. Animals received an oral administration of TMDZ or vehicle (distilled water) for 28 days, and then the blood was obtained, and hemorrheology parameters were detected. Values shown are mean ± SD, *n* = 10. TMDZ did not change any of the hemorrheology parameters after normal rats were administrated with TMDZ for 28 days.

Parameters	Control	Tianma Duzhong capsule		
		44.5 mg/kg	89 mg/kg	178 mg/kg
WBV: low shear (mPas) ∣ 10 s^−1^	9.86 ± 1.95	9.71 ± 1.56	10.16 ± 1.63	9.40 ± 1.58
WBV: moderate shear (mPas) ∣ 60 s^−1^	5.80 ± 0.97	5.61 ± 0.70	5.57 ± 0.41	9.03 ± 0.84
WBV: high shear (mPas) ∣ 150 s^−1^	4.54 ± 0.59	4.54 ± 0.48	4.63 ± 0.45	4.55 ± 0.67
PV	1.08 ± 0.03	1.06 ± 0.02	1.11 ± 0.04	1.09 ± 0.05
WBRV: low shear	21.36 ± 5.34	20.04 ± 3.77	17.62 ± 1.93	18.21 ± 2.79
WBRV: moderate shear	11.46 ± 2.17	10.54 ± 1.73	10.10 ± 0.92	10.24 ± 1.55
WBRV: high shear	8.39 ± 1.58	8.05 ± 1.18	7.93 ± 0.67	7.93 ± 1.19
Fb	2.10 ± 0.24	2.09 ± 0.17	2.19 ± 0.10	2.28 ± 0.43
HCT (%)	41.54 ± 2.11	43.34 ± 2.04	44.22 ± 3.22	43.30 ± 3.12
ESR(MM/H)	1.10 ± 0.18	1.10 ± 0.18	1.30 ± 0.67	2.30 ± 2.75
ESRK	3.77 ± 1.16	4.11 ± 1.23	4.29 ± 1.98	7.70 ± 8.23
RBCAI	2.15 ± 0.23	2.13 ± 0.17	2.40 ± 0.58	2.04 ± 0.25
RBCRI	7.79 ± 1.91	7.58 ± 1.41	7.70 ± 2.01	7.63 ± 1.32
RBCDI	1.05 ± 0.12	1.02 ± 0.09	1.06 ± 0.14	1.03 ± 0.09
RBCEI	5.21 ± 0.81	4.93 ± 0.54	5.93 ± 1.87	4.80 ± 0.69

WBV: whole blood viscosity; WBRV: whole blood reduced viscosity; PV: Plasma viscosity; Fb: fibrinogen; HCT: hematocrit; ESR: erythrocyte sedimentation rate; ESRK: erythrocyte sedimentation rate equation K value; RBCAI: red blood cell aggregation index; RBCRI: red blood cell rigidity index; RBCDI: red blood cell deformation index; RBCEI: red blood cell electrophoresis index.

**Table 3:** Hemorrheology parameters in stroke rats. Animals underwent 2 h MCAO and reperfusion and received either an oral administration of TMDZ or vehicle (distilled water) at 2 h MCAO, 4 h reperfusion, and every day later for 28 days, and then the blood was obtained, and hemorrheology parameters were detected. Values shown are mean ± S.D., *n* = 10. Statistical analysis was carried out with one-way ANOVA followed by a post hoc Tukey test.

Parameters	Sham	Ischemia/reperfusion control	Tianma Duzhong capsule		
			44.5 mg/kg	89 mg/kg	178 mg/kg
WBV: low shear (mPas) ∣ 10 s^−1^	9.08 ± 0.88	13.60 ± 3.10^##^	11.21 ± 2.10	8.75 ± 2.01**	9.14 ± 1.75**
WBV: moderate shear (mPas) ∣ 60 s^−1^	5.31 ± 0.37	6.74 ± 0.61^##^	5.95 ± 0.99*	5.08 ± 0.95**	5.22 ± 0.74**
WBV: high shear (mPas) ∣ 150 s^−1^	4.14 ± 0.41	5.26 ± 0.52^##^	4.67 ± 0.79	4.11 ± 0.77**	4.16 ± 0.47**
PV	1.04 ± 0.04	1.12 ± 0.06	1.12 ± 0.86	1.10 ± 0.06	1.07 ± 0.02*
WBRV: low shear	19.8 ± 2.98	28.18 ± 7.25^##^	25.02 ± 6.54	18.93 ± 3.04**	20.30 ± 4.27**
WBRV: moderate shear	10.53 ± 1.6	12.66 ± 1.59^##^	12.69 ± 4.75	9.99 ± 2.22**	10.40 ± 1.76**
WBRV: high shear	7.69 ± 1.74	9.30 ± 1.07^##^	8.66 ± 1.58	7.49 ± 1.56*	7.75 ± 1.08**
Fb	2.04 ± 0.26	2.90 ± 1.02^#^	2.64 ± 1.10	2.27 ± 0.32	2.10 ± 0.42*
HCT (%)	41.0 ± 4.41	44.61 ± 3.74	42.97 ± 6.71	40.14 ± 5.39	39.8 ± 3.30**
ESR(MM/H)	1.11 ± 0.33	2.90 ± 2.88	3.20 ± 4.96	1.38 ± 0.52	1.40 ± 0.52
ESRK	3.79 ± 1.46	10.54 ± 9.93	9.45 ± 9.20	4.26 ± 1.08	4.35 ± 1.44
RBCAI	2.22 ± 0.32	2.59 ± 0.51	2.41 ± 0.37	2.13 ± 0.21*	2.19 ± 0.27*
RBCRI	7.42 ± 1.89	8.29 ± 0.92	7.39 ± 1.21	6.83 ± 1.33*	7.22 ± 1.06*
RBCDI	1.05 ± 0.18	1.04 ± 0.08	1.02 ± 0.12	1.02 ± 0.13	1.05 ± 0.09
RBCEI	5.43 ± 0.70	5.86 ± 1.34	5.69 ± 0.92	5.35 ± 0.57	5.53 ± 0.86

^#^ *P* < 0.05 and ^##^ *P* < 0.01 versus sham-operated group (Sham); *P < 0.05 and **P < 0.01 versus ischemic-reperfusion control group (IR Control). WBV: whole blood viscosity; WBRV: whole blood reduced viscosity; PV: Plasma viscosity; Fb: fibrinogen; HCT: heamatocrit; ESR: erythrocyte sedimentation rate; ESRK: erythrocyte sedimentation rate equation K value; RBCAI: red blood cell aggregation index; RBCRI: red blood cell rigidity index; RBCDI: red blood cell deformation index; RBCEI: red blood cell electrophoresis index.

## References

[1] Mohammad YM, Divani AA, Kirmani JF, Harris-Lane P, Qureshi AI (2004). Acute treatment for ischemic stroke in 2004. *Emergency Radiology*.

[2] Cheng YD, Al-Khoury L, Zivin JA (2004). Neuroprotection for Ischemic Stroke: two decades of success and failure. *NeuroRx*.

[3] Lee M-S, Yang D-Y, Cheng C-L, Liang Y-J, Yang L-L, Cheng F-C (2003). Ginkgo biloba extract preserves pyruvate and enhances ascorbate in the cortex of gerbils during focal cerebral ischemia: a microdialysis-liquid chromatography study. *Journal of Chromatography A*.

[4] Cao X-Q, Zhang X-M, Wei X-B, Wang L-X, Liu H-Q (2002). Protective effects of gypenosides on focal brain ischemia-reperfusion injury in rats. *Chinese Pharmaceutical Journal*.

[5] Hong JT, Ryu SR, Kim HJ (2001). Protective effect of green tea extract on ischemia/reperfusion-induced brain injury in Mongolian gerbils. *Brain Research*.

[6] Yan B, Wang D-Y, Xing D-M (2004). The antidepressant effect of ethanol extract of radix puerariae in mice exposed to cerebral ischemia reperfusion. *Pharmacology Biochemistry and Behavior*.

[7] Numagami Y, Sato S, Ohnishi ST (1996). Attenuation of rat ischemic brain damage by aged garlic extracts: a possible protecting mechanism as antioxidants. *Neurochemistry International*.

[8] Zhang H-L, Gu Z-L, Savitz SI, Han F, Fukunaga K, Qin Z-H (2008). Neuroprotective effects of prostaglandin A1 in rat models of permanent focal cerebral ischemia are associated with nuclear factor-*κ*B inhibition and peroxisome proliferator-activated receptor-*γ* up-regulation. *Journal of Neuroscience Research*.

[9] Zhang RL, Chopp M, Zhang ZG, Jiang Q, Ewing JR (1997). A rat model of focal embolic cerebral ischemia. *Brain Research*.

[10] Schallert T, Fleming SM, Leasure JL, Tillerson JL, Bland ST (2000). CNS plasticity and assessment of forelimb sensorimotor outcome in unilateral rat models of stroke, cortical ablation, parkinsonism and spinal cord injury. *Neuropharmacology*.

[11] Haelewyn B, Freret T, Pacary E (2007). Long-term evaluation of sensorimotor and mnesic behaviour following striatal NMDA-induced unilateral excitotoxic lesion in the mouse. *Behavioural Brain Research*.

[12] Brenneman M, Sharma S, Harting M (2010). Autologous bone marrow mononuclear cells enhance recovery after acute ischemic stroke in young and middle-aged rats. *Journal of Cerebral Blood Flow and Metabolism*.

[13] Neumann-Haefelin T, Kastrup A, de Crespigny A (2000). Serial MRI after transient focal cerebral ischemia in rats: dynamics of tissue injury, blood-brain barrier damage, and edema formation. *Stroke*.

[14] Yoneda K, Iwamura R, Kishi H, Mizukami Y, Mogami K, Kobayashi S (2004). Identification of the active metabolite of ticlopidine from rat in vitro metabolites. *British Journal of Pharmacology*.

[15] Belayev L, Alonso OF, Busto R, Zhao W, Ginsberg MD (1996). Middle cerebral artery occlusion in the rat by intraluminal suture: neurological and pathological evaluation of an improved model. *Stroke*.

[16] Belayev L, Khoutorova L, Atkins K, Cherqui A, Alvarez-Builla J, Bazan NG (2009). LAU-0901, a novel platelet-activating factor receptor antagonist, confers enduring neuroprotection in experimental focal cerebral ischemia in the rat. *Brain Research*.

[17] Wang Y, Zhang ZG, Rhodes K (2007). Post-ischemic treatment with erythropoietin or carbamylated erythropoietin reduces infarction and improves neurological outcome in a rat model of focal cerebral ischemia. *British Journal of Pharmacology*.

[18] Kowal P, Marcinkowska-Gapińska A (2007). Hemorheological changes dependent on the time from the onset of ischemic stroke. *Journal of the Neurological Sciences*.

[19] Velcheva I, Nikolova G (2008). Hemorheological disturbances and cognitive function in patients with cerebrovascular disease. *Clinical Hemorheology and Microcirculation*.

[20] Kuroda S, Siesjö BK (1997). Reperfusion damage following focal ischemia: pathophysiology and therapeutic windows. *Clinical Neuroscience*.

[21] Abumiya T, Fitridge R, Mazur C (2000). Integrin *α*(IIb)*β*3 inhibitor preserves microvascular patency in experimental acute focal cerebral ischemia. *Stroke*.

[22] Choudhri TF, Hoh BL, Zerwes H-G (1998). Reduced microvascular thrombosis and improved outcome in acute murine stroke by inhibiting GP IIb/IIa receptor-mediated platelet aggregation. *Journal of Clinical Investigation*.

